# Supplementary material for: Obese Vegetarians and Omnivores Show Different Metabolic Changes: Analysis of 1340 Individuals
Source: Nutrients. 2022 May 26;14(11):2204. doi: 10.3390/nu14112204 (PMC9182907; doi:10.3390/nu14112204)
Supplement: Supplementary file 1 [file nutrients-14-02204-s001.zip › nutrients-1718049-supplementary.pdf]

Supplementary material

Table S1. Statistics and p-values of comparisons between omnivores and vegetarians for biochemical markers of inflammation, liver function, and insulin resistance in menstruating and non-menstruating obese men and women (BMI $\geq$ 30 kg/m^2)

Variable	Obese men		Obese women that do not menstruate		Obese women that menstruate	
	F-test	*p*Value	F-test	*p*Value	F-test	*p*Value
hs-CRP	$F_{(1;52)}$ = 2.34	0.1321	$F_{(1;25)}$ = 1.75	0.1984	$F_{(1;47)}$ = 0.11	0.7421
HOMAIR	$F_{(1;56)}$ = 0.08	0.7851	$F_{(1;29)}$ = 0.40	0.5326	$F_{(1;57)}$ = 2.87	0.0955
ALT	$F_{(1;56)}$ = 3.07	0.0854	$F_{(1;29)}$ = 1.02	0.3200	$F_{(1;60)}$ = 1.45	0.2327
AST	$F_{(1;56)}$ = 1.76	0.1906	$F_{(1;29)}$ = 0.17	0.6838	$F_{(1;60)}$ = 0.26	0.6123
GGT	$F_{(1;55)}$ = 10.51	**0.0020**	$F_{(1;29)}$ = 5.42	**0.0271**	$F_{(1;57)}$ = 4.44	**0.0395**
Ferritin	$F_{(1;56)}$ = 40.44	**< 0.0001**	$F_{(1;30)}$ = 10.15	**0.0034**	$F_{(1;58)}$ = 6.92	**0.0109**
HBA1C	$F_{(1;55)}$ = 0.17	0.6815	$F_{(1;29)}$ = 0.25	0.6183	$F_{(1;58)}$ = 1.58	0.1807

Significant differences between omnivores and vegetarians are highlighted in bold.
